# WSVAD-CLIP: Temporally Aware and Prompt Learning with CLIP for Weakly Supervised Video Anomaly Detection

**DOI:** 10.3390/jimaging11100354

**Published:** 2025-10-10

**Authors:** Min Li, Jing Sang, Yuanyao Lu, Lina Du

**Affiliations:** School of Artificial Intelligence and Computer Science, North China University of Technology, No. 5 Jinyuanzhuang Road, Beijing 100144, China; sj15137183524@163.com (J.S.); luyy@ncut.edu.cn (Y.L.); 16650393348@163.com (L.D.)

**Keywords:** WSVAD-CLIP, CLIP, Axial-Graph Module, Text Prompt Mechanism, Video Anomaly Detection

## Abstract

Weakly Supervised Video Anomaly Detection (WSVAD) is a critical task in computer vision. It aims to localize and recognize abnormal behaviors using only video-level labels. Without frame-level annotations, it becomes significantly challenging to model temporal dependencies. Given the diversity of abnormal events, it is also difficult to model semantic representations. Recently, the cross-modal pre-trained model Contrastive Language-Image Pretraining (CLIP) has shown a strong ability to align visual and textual information. This provides new opportunities for video anomaly detection. Inspired by CLIP, WSVAD-CLIP is proposed as a framework that uses its cross-modal knowledge to bridge the semantic gap between text and vision. First, the Axial-Graph (AG) Module is introduced. It combines an Axial Transformer and Lite Graph Attention Networks (LiteGAT) to capture global temporal structures and local abnormal correlations. Second, a Text Prompt mechanism is designed. It fuses a learnable prompt with a knowledge-enhanced prompt to improve the semantic expressiveness of category embeddings. Third, the Abnormal Visual-Guided Text Prompt (AVGTP) mechanism is proposed to aggregate anomalous visual context for adaptively refining textual representations. Extensive experiments on UCF-Crime and XD-Violence datasets show that WSVAD-CLIP notably outperforms existing methods in coarse-grained anomaly detection. It also achieves superior performance in fine-grained anomaly recognition tasks, validating its effectiveness and generalizability.

## 1. Introduction

Video Anomaly Detection (VAD) aims to automatically detect anomalous patterns or events that deviate from normal behavior in video sequences [1,2,3], holding significant value in practical applications such as public safety. As anomalous behaviors are both uncommon and diverse in nature, the detection task faces considerable challenges [4]. Effective VAD technology can assist intelligent surveillance systems in promptly detecting potential threats, thereby enhancing public safety [5,6].

Early VAD methods primarily adopted one-class classification approaches (semi-supervised) [7,8,9], but they often misclassified unseen normal behaviors as anomalies. Fully supervised methods require frame-level annotations [10,11], which are prohibitively expensive, while unsupervised methods usually suffer from suboptimal performance due to a lack of explicit supervision [12]. In contrast, Weakly Supervised Video Anomaly Detection (WSVAD) has become a practical compromise, requiring only video-level labels while maintaining competitive detection performance [5,13,14,15,16]. Recently, many researchers have focused on weakly supervised VAD.

Most WSVAD methods extract frame-level features using pre-trained models such as Convolutional 3D Network (C3D) [5,17], Inflated 3D ConvNet (I3D) [3,18], and Vision Transformer (ViT) [19,20], followed by Multiple Instance Learning (MIL) to predict anomaly scores. Despite their progress, two major challenges remain: modeling temporal dependencies of anomalies with diverse durations and capturing the complex semantics of anomalies from weak supervision [15,16].

In the past two years, Large Language and Vision (LLV) models and Vision-Language Pretraining (VLP) models [21,22,23,24] such as CLIP [25] have achieved deep alignment between vision and language modalities, providing new opportunities to leverage textual semantics for anomaly detection.

Recently, several studies have attempted to leverage CLIP for video anomaly detection. However, existing methods still show clear gaps: some rely only on visual features from CLIP’s encoder [26,27], while others fail to effectively model vision-language relationships [28]. Consequently, they cannot fully exploit CLIP’s cross-modal capability for anomaly understanding.

To address these limitations, this paper introduces WSVAD-CLIP, a framework that enhances both temporal modeling and semantic alignment. Specifically, an Axial-Graph (AG) Module is proposed, which combines Lite Graph Attention Networks (LiteGAT) [29] and an Axial Transformer [30] to capture both local motion variations and long-range dependencies. Furthermore, unlike prior works that only use CLIP’s original text embeddings, a Text Prompt is designed to integrate learnable prompt [31] with knowledge-enhanced prompt [32], and it is further extended with an Abnormal Visual-Guided Text Prompt (AVGTP) [33] to inject abnormal visual cues into textual embeddings. These designs enable richer semantic representations and stronger cross-modal alignment for anomaly detection.

The main contributions of this work are summarized as follows:

(1) A temporal modeling AG Module is introduced that simultaneously captures temporal dependencies through an Axial Transformer and LiteGAT, effectively enhancing the performance of the WSVAD task.

(2) A Text Prompt mechanism is proposed that combines learnable prompt with knowledge-enhanced prompt to gain deeper insights into the specific semantics of video anomalies. Visual information and text are further integrated through an AVGTP mechanism to generate more informative class-level embeddings.

(3) Extensive experiments are conducted on two representative large-scale benchmark datasets, UCF-Crime [5] and XD-Violence [3], and the results demonstrate the superior performance of the proposed framework.

## 2. Related Work

### 2.1. Video Anomaly Detection

Under the current research paradigm, video anomaly detection tasks are commonly classified into four categories according to existing supervision methods. The first category is one-class classification, i.e., semi-supervised video anomaly detection, where the training process involves only normal instances [7,8,9,34]. The second category is fully supervised video anomaly detection, which requires detailed frame-level annotations in the training set [10,11]. The third category is unsupervised video anomaly detection, which identifies anomalies from completely unlabeled videos [12]. The fourth category is weakly supervised video anomaly detection, which is trained with video-level normal/abnormal annotations [5,13,14,15,16,35,36,37].

Among these methods, weakly supervised video anomaly detection has attracted growing attention in the last few years, becoming a research hotspot. WSVAD generally achieves encouraging performance while reducing annotation costs. Sultani et al. pioneered the formulation of WSVAD as a Multiple Instance Learning (MIL) task [5]. Each video was regarded as a bag, with its temporal segments considered as instances. By leveraging a video-level ranking loss, their model aimed to maximize the separation between the most anomalous instances in positively and negatively labeled video bags. Subsequently, Zhong et al. proposed a method based on Graph Convolutional Network (GCN) to capture the similarity of features and temporal consistency between video segments [38]. Tian et al. designed a robust temporal feature magnitude learning strategy to enhance the robustness of MIL-based methods against negative instances within anomalous videos, and integrated dilated convolutions and self-attention mechanisms to capture both long-range and short-range temporal dependencies [16]. Li et al. developed a Multi-Sequence Learning (MSL) model based on the Transformer architecture to estimate anomaly likelihoods at both the video and segment levels [20]. Zhou et al. proposed an Uncertainty-Regulated Dual-Memory Unit (UR-DMU) to distinguish between normal and anomalous instances, and embedded global and local self-attention strategies within a Transformer framework to effectively capture temporal dependencies [39]. The aforementioned methods focus solely on coarse-grained VAD. Wu et al. proposed a fine-grained WSVAD approach that differentiates between various types of anomalous frames [40], this approach also enables anomaly detection using multimodal information. Current WSVAD methods focus solely on visual patterns, neglecting the rich semantic information that could be obtained through vision-language models.

### 2.2. Large Language and Vision Models

Recently, large-scale pre-trained models have made significant advancements, Zanella et al. designed LAVAD [41], which leverages pre-trained large language models and vision-language models to perform anomaly detection without any training by generating and analyzing textual descriptions of video frames. Dev et al. proposed MCANet, a training-free multi-modal framework leveraging vision, audio, and language models to generate captions for anomaly detection without extra data or retraining [42]. CLIP is also one of the most representative models This model has demonstrated unprecedented performance in various image-related tasks such as image classification [31], object detection [43], and semantic segmentation [44]. Lately, CLIP has also been effectively adapted to the video field. For instance, VideoCLIP was designed to align video–text representations via contrasting time-overlapping video–text pairs with extracted hard negative samples [45]. Some recent works have also explored the application of VAD using CLIP. Joo et al. proposed leveraging CLIP to effectively extract discriminative visual features; however, their approach did not utilize textual information [26].

Zanella et al. employed a feature space transformation based on CLIP to learn anomalies and utilized semantic information to perform MIL segment selection and anomaly identification [28]. Yang et al. introduced TPWNG, which fuses visual features with textual features derived from CLIP’s text encoder to produce pseudo labels, and then adopts a supervised learning approach for anomaly training [46]. The OPVAD method proposed by Wu et al. addresses a more realistic and open setting for video anomaly detection [47]. The above three studies all adopt CLIP-based approaches that integrate visual and textual modalities for anomaly detection, including multimodal transformer-based methods. Existing methods that leverage textual information remain relatively simplistic. To address these limitations, this work proposes a novel approach that more deeply integrates the vision-language knowledge of the pre-trained CLIP model, enhancing the performance of weakly supervised video anomaly detection and enabling finer-grained recognition of abnormal events.

## 3. Method

In this section, the proposed WSVAD approach is presented. First, the overall architecture is introduced, followed by a detailed explanation of its core components.

### 3.1. Overall Architecture

The WSVAD task aims to train a model for frame-level anomaly detection using data that only contains video-level labels. In this task, the training data consists of several tuples (V, y), where V represents a complete video, and y is a binary label denoting the presence or absence of anomalous frames in V. If all frames in the video are normal, the label y is 0; if there exists at least one abnormal frame, the label y is 1.

**Figure 1:**
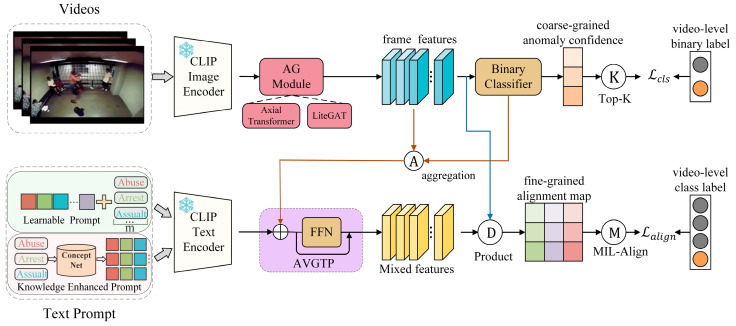
The overall architecture of WSVAD-CLIP. The framework includes the CLIP image encoder for extracting visual features, the text prompt module for generating learnable prompt and knowledge-enhanced prompt, the text encoder for producing semantic embeddings, and the AG module for temporal modeling. The AVGTP mechanism fuses abnormal visual information with textual features to form hybrid representations, enabling fine-grained anomaly recognition.

Our framework, as illustrated in Figure 1, consists of both abnormal and normal training videos being fed into the CLIP image encoder to obtain visual features. The anomaly category labels are processed through learnable prompt and knowledge-enhanced prompt, respectively, and then passed through the text encoder to obtain fused anomaly feature embeddings. To capture temporal dependencies, the visual features are input into the AG Module to produce frame-level representations. These representations are then used for coarse-grained anomaly detection through training a binary classifier. Furthermore, The AVGTP combines the aggregated abnormal visual information with the fused textual features to form hybrid prompt features. These hybrid features are then aligned with visual frame features at a fine-grained level, enabling fine-grained abnormal event recognition [33].

### 3.2. AG Module

**Figure 2:**
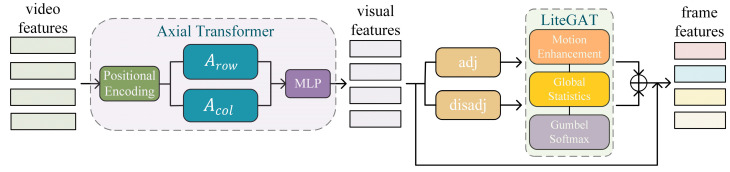
The architecture of AG Module. Video features are processed by the Axial Transformer to capture global temporal dependencies, then modeled with dual-branch LiteGAT using an adjacency graph (adj) and a distance graph (disadj), and finally fused to obtain enhanced frame-level representations.

In the WSVAD task, video frames are fed into the CLIP image encoder to obtain video features. However, to bridge the gap between CLIP and video-based tasks, it is essential to model the temporal dependencies inherent in videos. To address this issue, a novel AG Module is proposed that captures both global and local temporal information by integrating LiteGAT with an Axial Transformer [29,30]. The architecture of the AG Module is illustrated in Figure 2.

The AG Module first feeds video features into the Axial Transformer, where positional encoding and dual-axis attention are employed to model the global temporal dependencies between frames, resulting in initial visual features. Then, two graph structures are constructed: an adjacency graph (adj) based on semantic similarity and a distance graph (disadj) based on temporal intervals. These are fed into the dual-branch LiteGAT module. Each branch incorporates motion enhancement, global statistics modeling, and Gumbel Softmax sampling implemented using PyTorch 2.5.1 to capture structural features between keyframes at both local and global levels. Finally, the outputs of both branches are fused with the Axial Transformer features to enhance the expressiveness of frame-level representations.

#### 3.2.1. Axial Transformer

To enhance the temporal modeling capability between frame-level features, the AxialTransformerWithMLP module is introduced [30], which leverages an axial attention mechanism to model dependencies along the row and column dimensions of the spatial layout independently. Given an input feature tensor $F\in R^{B\times C\times H\times W}$, where B denotes the batch size, C is the channel dimension, and H×W represents the temporal-spatial layout, first, a learnable positional encoding $P\in R^{1\times H\times W\times C}$ is incorporated to enhance the model’s positional awareness. By adding it to the original input features, the position-enhanced feature $F_{pe}$ is obtained, as shown in Equation (1):

(1) $$F_{pe}=F+P$$

Subsequently, the feature map is passed through two consecutive axial attention modules, which perform self-attention operations along the row and column dimensions, respectively. Let $A_{row}$ and $A_{col}$ denote the outputs of the row-wise and column-wise attention modules. The two are fused to obtain the attention-enhanced feature $F_{attn}$, as shown in Equation (2):

(2) $$F_{attn}=A_{row}+A_{col}$$

To further enhance the model’s capability in capturing complex temporal patterns, a Multilayer Perceptron (MLP) is introduced following the attention modules, combined with layer normalization (LayerNorm, LN) and residual connection mechanisms. The final output feature $F_{out}$ is obtained, as shown in Equation (3):

(3) $$F_{out}=F_{attn}+MLP\left(LN\left(F_{attn}\right)\right)$$

This module effectively integrates both local and global temporal dependencies, enhancing the feature representations’ sensitivity to abnormal dynamics. As a result, it facilitates more accurate identification of potential anomalous segments in WSVAD.

#### 3.2.2. LiteGAT

To enhance the modeling of abnormal correlations among frame-level features, LiteGAT [29] is proposed, designed to effectively model local and global relationships within weakly supervised video anomaly detection. Specifically, LiteGAT takes as input a sequence of frame-level features $F_{out}\in R^{T\times d}$ produced by the preceding stage (e.g., Axial Transformer). It first applies linear transformations to generate the Query (Q), Key (K), and Value (V) matrices, and an attention map is constructed based on the scaled dot-product mechanism. To improve the model’s capacity to attend to potential abnormal regions, a motion-aware mechanism is introduced. The L2 norm of the frame features is calculated to obtain a motion intensity vector $m\in R^{T}$, which is then mapped via an MLP to a motion bias matrix $B\in R^{T\times T}$ and added to the attention scores. The final attention calculation is shown in Equation (4):

(4) $$Attention\left(Q,K,V\right)=Softmax\left(\frac{QK^{T}}{\sqrt{d}}+B\right)V$$

The elements in B reflect the motion differences between frames, thereby guiding the attention mechanism to focus more on regions with significant motion variations, which enhances the sensitivity to anomalies. Furthermore, to promote sparsity and discriminative capacity within the attention structure, Gumbel Softmax sampling is introduced during training to perturb the original attention map and construct a sparse adjacency structure. Let $A_{ij}$ denote the original attention scores; the perturbed attention map ${\tilde{A}}_{ij}$ is then defined as Equation (5):

(5) $${\tilde{A}}_{ij}=\frac{\exp\left(\left(\log A_{ij}+g_{ij}\right)/\tau \right)}{\sum_{k}\exp\left(\left(\log A_{ik}+g_{ik}\right)/\tau \right)}$$

Here, $g_{ij}$ represents noise sampled from a Gumbel distribution, the temperature coefficient τ is adopted to regulate the smoothness of sampling. This mechanism effectively guides the model to focus on a small number of key inter-frame connections, enhancing its ability to discriminate abnormal segments. Furthermore, to balance local modeling capability and global feature stability, a residual fusion mechanism is introduced that constructs a global modulation path $R\in R^{T\times d}$ based on the mean and standard deviation statistics of the input features. This path is fused with the attention-aggregated intermediate features $F^{\prime }\in R^{T\times d}$, resulting in the final output features $F_{out}^{\prime }$, as shown in Equation (6):

(6) $$F_{out}^{\prime }=\alpha \cdot F^{\prime }+\left(1-\alpha \right)\cdot R$$

Here, $\alpha \in \left[0,1\right]$ is a learnable fusion coefficient that represents the weight balance between local attention and global statistics. By incorporating motion bias enhancement, sparse attention sampling, and global statistical fusion, the proposed LiteGAT module effectively models temporal abnormal dependencies in videos. This design improves the accuracy and robustness of anomaly localization under weak supervision without relying on frame-level labels.

### 3.3. Text Prompt

For weakly supervised video anomaly detection tasks, raw textual labels (e.g., “fighting”, “accident”) are often overly simplistic and insufficient to comprehensively describe complex anomalous events. To overcome this limitation, a Text Prompt mechanism is proposed that integrates learnable prompt and knowledge-enhanced prompt, constructing more expressive textual representations through contextual expansion and external semantic enrichment [31,32].

#### 3.3.1. Learnable Prompt

In this study, to enhance the expressiveness of label texts and improve cross-modal alignment, a Learnable Prompt mechanism is introduced. Inspired by the Context Optimization for Prompting (CoOp) method, the original discrete category labels are augmented with a set of learnable context vectors, enabling more adaptive and expressive text inputs [31].

Specifically, a category label (e.g., “fighting”, “accident”) is first tokenized into a category token using the CLIP tokenizer. It is then concatenated with n learnable prompt vectors $\left\{c_{1},\ldots ,c_{n}\right\}$ on both sides, forming a complete prompt sequence that is fed into the text encoder as input $t_{prompt}$, as shown in Equation (7):

(7) $$t_{prompt}=\left\{c_{1},\ldots ,c_{n},t_{label},c_{n+1},\ldots ,c_{2n}\right\}$$

Here, $t_{label}$ denotes the original category token. In this manner, the category token is placed at the center of the sequence. The resulting input sequence, along with the learnable prompts, is then fed into the CLIP text encoder, where it undergoes positional encoding to produce the final category embedding $t_{out}\in R^{d}$.

#### 3.3.2. Knowledge-Enhanced Prompt

To enhance the semantic expressiveness of category labels and improve alignment between textual and visual modalities, a Knowledge-Enhanced Prompt mechanism is introduced based on a knowledge graph [32]. Specifically, ConceptNet is leveraged as an external knowledge source to retrieve semantically related concept words for each category label, which are then incorporated as additional contextual information.

Formally, given a category label c, all concept words associated with it are first retrieved from ConceptNet based on a pre-defined set of semantic relations, forming its corresponding concept set $k_{c}$, as shown in Equation (8):

(8) $$k_{c}=\left\{k_{1},k_{2},..,{k_{i},..,k}_{m}\right\}$$

Each $k_{i}$ represents a node in the semantic graph that is closely connected to the category c. The concepts are first ranked based on the confidence scores of their associated edges, and only those with scores greater than 0.6 are retained. From this filtered set, the top-6 high-quality concepts are selected to form the final knowledge-enhanced prompt. These concept words are then individually encoded using the CLIP text encoder to obtain their embedding representations. Finally, the embeddings are averaged to produce the knowledge-enhanced prompt representation $t_{c}^{know}$ for category c, as shown in Equation (9):

(9) $$t_{c}^{know}=\frac{1}{M}\sum_{i=1}^{M}{CLIP}_{text}\left(k_{i}\right)$$

This representation captures not only the explicit semantics of the category label but also incorporates implicit semantic relations from the knowledge graph, thereby enhancing the discriminative power of the category embedding. It provides richer prior knowledge to support cross-modal anomaly detection. Finally, a linear weighted fusion of the learnable prompt and the knowledge-enhanced prompt is performed at the feature level to construct a unified textual representation with improved generalization capability.

### 3.4. Abnormal Visual-Guided Text Prompt

To enhance the semantic expressiveness of textual labels in representing anomalous events, visual context information is introduced to dynamically refine category embeddings. Compared to static textual descriptions, visual features offer more discriminative contextual cues, particularly in abnormal segments. To this end, an Abnormal Visual-Guided Text Prompt (AVGTP) is proposed [33], which focuses on extracting key visual features from anomalous segments and aggregating them into a video-level prompt signal. This prompt is then fused with the original text prompt to achieve fine-grained optimization of the category embedding.

Specifically, the anomaly confidence scores A obtained from coarse-grained anomaly detection are initially treated as anomaly attention, which is then used to compute the video-level prompt via a dot product with the frame features $F_{out}^{\prime }$, as shown in Equation (10):

(10) $$V_{attn}=Norm\left(A^{T}F_{out}^{\prime }\right)$$

Here, Norm denotes a normalization operation, and $V_{attn}\in R^{d}$ represents the abnormal visual-guided prompt. $V_{attn}$ is then added to the category embedding $t_{text}$, and pass the result through a simple Feed-Forward Network (FFN) [48], followed by a skip connection, to generate the final instance-specific category embedding T, as shown in Equation (11):

(11) $$T=FFN\left(ADD\left(V_{attn},t_{text}\right)\right)+t_{text}$$

Here, ADD denotes element-wise addition. Through this process, the category embedding is able to incorporate visual contextual information from the anomalous segments of the current video, thereby enhancing its adaptability to abnormal semantics.

Finally, the updated category embedding T is used to compute a similarity-based alignment map M with the frame-level features $F_{out}^{\prime }$, which serves as the foundation for subsequent anomaly category recognition.

### 3.5. Objective Function

To effectively distinguish abnormal events in videos, a multi-task training objective is designed, which consists of classification loss, semantic alignment loss, and contrastive loss. These components collaboratively optimize the model’s capacity to express abnormal semantics.

First, for video-level supervision, a Top-K strategy is used that selects the top K scoring frames from videos labeled as normal and abnormal, using their scores as the final video-level prediction. The binary cross-entropy loss is employed for quantifying the discrepancy between the predicted results and the ground truth labels, $L_{cls}$ as shown in Equation (12):

(12) $$L_{cls}=BCE\left(\hat{y,}y\right)$$

Here, $\hat{y}$ denotes the predicted video-level score from the model, and y represents the corresponding ground truth label.

Furthermore, to address the challenge of category alignment under weak supervision, a Multiple Instance Learning Alignment (MIL-Align) mechanism is introduced. Specifically, an alignment matrix M is constructed between frame-level visual features and all category embeddings. For each category, the top k similarity scores in the corresponding column of M are selected and their average is computed, yielding the alignment score $s_{i}$ between the current video and the i-th category. The set of scores for all categories is represented as a vector $S=\left\{s_{1},s_{2},\ldots ,s_{m}\right\}$, where m represents the total number of categories. Each video is expected to have the highest similarity with its corresponding text label. To this end, the softmax function is applied to compute the predicted probability $p_{i}$ for each category, as shown in Equation (13):

(13) $$p_{i}=\frac{exp\left(s_{i}/\tau \right)}{\sum_{j}exp\left(s_{j}/\tau \right)}$$

Here, τ is a temperature scaling factor. Based on this, the cross-entropy loss is employed for quantifying the discrepancy between the ground-truth category and the predicted distribution, and define the semantic alignment loss $L_{align}$ as shown in Equation (14):

(14) $$L_{align}=-\log\left(p_{y}\right)$$

Here, $p_{y}$ denotes the predicted probability corresponding to the ground-truth category.

To further enhance the discriminability among category embeddings, a contrastive loss $L_{contrast}$ is introduced. This loss aims to push the normal category embedding away from all abnormal category embeddings in the semantic space. Cosine similarity is adopted as the similarity measure, as shown in Equation (15):

(15) $$L_{contrast}=\sum_{j}max\left(0,\frac{t_{n}^{T}\cdot t_{aj}}{{\left\|t_{n}\right\|}_{2}\cdot {\left\|t_{aj}\right\|}_{2}}\right)$$

Here, $t_{n}$ denotes the embedding of the normal category, and $t_{aj}$ denotes the embedding of the *j*-th abnormal category. This loss penalizes excessively high similarity scores to enlarge the semantic distance between normal and anomalous categories.

In summary, our final loss function $L_{total}$ is composed of the three aforementioned components, as shown in Equation (16):

(16) $$L_{total}=L_{cls}+L_{align}+{\lambda L}_{contrast}$$

Here, λ is a weighting coefficient used to balance the contribution of the contrastive loss.

## 4. Experiments

### 4.1. Datasets and Evaluation Metrics

#### 4.1.1. Datasets

**Figure 3:**
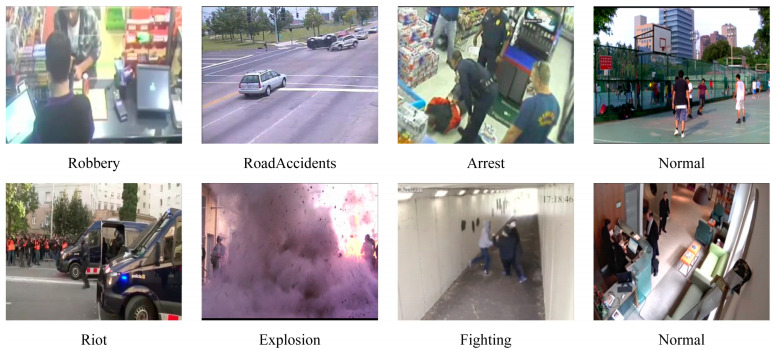
Example video frames from the UCF-Crime and XD-Violence datasets, including both abnormal and normal events.

The study is conducted on two widely adopted benchmarks for weakly supervised video anomaly detection: UCF-Crime and XD-Violence [3,5]. Example video frames from the two datasets are shown in Figure 3. UCF-Crime is a large-scale real-world surveillance video dataset for WSVAD. It contains a total of 128 h of video, including 1900 surveillance clips that cover 13 real-world anomaly categories that pose major risks to public safety. Specifically, the dataset includes Abuse, Arrest, Arson, Assault, Burglary, Explosion, Fighting, RoadAccidents, Robbery, Shooting, Shoplifting, Stealing, and Vandalism. A notable characteristic of UCF-Crime is that most videos begin with normal segments before transitioning into abnormal events. Among them, 1610 videos are used for training and 290 for testing. XD-Violence is a comprehensive dataset for large-scale violence detection, sourced from diverse media such as films, online content, surveillance videos, and CCTV footage. It comprises 4754 video clips totaling 217 h, encompassing six categories of abnormal events: abuse, car accidents, explosions, fights, riots, and shootings. To prevent violence detection systems from relying on scene background rather than actual events, XD-Violence additionally collects a large number of non-violent videos that share similar backgrounds with violent ones. Out of these, 3954 clips are designated for training, while the remaining 800 are used for testing.

UCF-Crime and XD-Violence provide valuable resources for weakly supervised video anomaly detection, but both datasets also exhibit certain class imbalances and potential data biases. In UCF-Crime, the Burglary and Stealing categories each contain 100 videos, and RoadAccidents and Robbery each contain 150 videos; these four categories correspond to relatively common real-world anomalies, while the remaining nine categories each have only 50 videos. In XD-Violence, the number of video segments per category also varies, for example, Fighting has 2363 segments, Shooting 1845 segments, Abuse 1290 segments, Car Accidents 1262 segments, Explosion 1101 segments, and Riot only 981 segments, reflecting the difference between more common and less common events. Furthermore, XD-Violence is a multi-label dataset, as a single video may contain multiple types of violent behavior, which further increases the complexity of the data distribution.

#### 4.1.2. Evaluation Metrics

Binary WSVAD, i.e., video-level abnormal vs. normal detection, is referred to as coarse-grained anomaly detection. Following previous works, frame-level Area Under the Curve (AUC) and anomalous video-level AUC (AnoAUC) are reported on UCF-Crime, and frame-level Average Precision (AP) on XD-Violence. For fine-grained WSVAD, i.e., identifying specific abnormal categories, it is referred to as fine-grained anomaly recognition. The conventional evaluation protocol from video action detection is employed, using mean Average Precision (mAP) calculated at various Intersection over Union (IoU) thresholds. In this work, mAP is computed using IoU thresholds ranging from 0.1 to 0.5 with a step size of 0.1, which is a commonly adopted evaluation strategy in weakly supervised video anomaly detection. The average mAP (AVG) across these thresholds is reported as the main performance metric to ensure robustness and comparability. The range of 0.1 to 0.5 is chosen because event boundaries in anomaly detection often involve considerable uncertainty; excessively high thresholds may be overly strict, while overly low thresholds may fail to reflect localization accuracy, making this interval a balanced choice. Note that mAP is calculated only on the abnormal videos in the test set.

### 4.2. Implementation Details

For the visual and textual encoders in our network, the pretrained CLIP model (ViT-B/16) is adopted and kept frozen during training. The FFN module refers to the standard feed-forward block from the Transformer architecture. The visual input dimensions are set to 256 in length and 512 in width. The fusion weight α in Equation (6) is set to 0.7, and the temperature factor τ in Equation (13) to 0.07. The context length l is set to 20. The coefficient λ in Equation (16) is set to 1 × 10^−1^ for UCF-Crime and 1 × 10^−4^ for XD-Violence. For the knowledge-enhanced prompting, a confidence threshold of 0.7 is applied to filter low-relevance concepts. All experiments are conducted on a single NVIDIA GeForce RTX 2080 Ti GPU, with the random seed fixed at 234 to ensure reproducibility. The model is optimized using the AdamW optimizer with a batch size of 64. Gradient clipping with a maximum norm of 1.0 is applied during training. For UCF-Crime, the weight decay is set to 0.02, the learning rate to 5 × 10^−5^, and the model is trained for 10 epochs, which takes approximately 7 h. For XD-Violence, the weight decay is set to 0.01, the learning rate to 2 × 10^−5^, and the model is trained for 10 epochs, which takes approximately 15 h.

### 4.3. Comparison with State-of-the-Art Methods

The method’s performance is evaluated on the UCF-Crime and XD-Violence datasets against the latest state-of-the-art (SOTA) approaches. Specifically, the WSVAD-CLIP model is evaluated against recent advanced methods in both coarse-grained anomaly detection and fine-grained anomaly recognition tasks. The following sections present detailed results and analysis.

As shown in Table 1 and Table 2, competitive results are yielded on both the UCF-Crime and XD-Violence datasets for coarse-grained anomaly detection by WSVAD-CLIP. Specifically, an AUC of 87.85% on UCF-Crime, significantly outperforming methods that do not leverage CLIP features (comparison is made with UR-DMU) [39] and showing clear advantages over other CLIP-based approaches (comparisons are performed against both CLIP-TSA and TPWNG) [26,46]. While the improvement over TPWNG is relatively modest at 0.06%, this is still a noteworthy gain given the maturity of that method. Compared to CLIP-TSA and UR-DMU, our method achieves absolute gains of 0.27% and 0.88%, respectively. On the XD-Violence dataset, improved AP performance is also shown, outperforming TPWNG by 0.27%. Furthermore, WSVAD-CLIP achieves absolute gains of 1.78% over CLIP-TSA and 2.29% over UR-DMU. These results across two large-scale benchmark datasets highlight the strong generalization ability and effectiveness of the method for WSVAD.

The ShanghaiTech dataset was employed as an auxiliary benchmark to preliminarily assess the cross-scenario generalization capability of the proposed model [7]. This dataset focuses on anomalous pedestrian behaviors in a campus environment, where the test set contains normal videos alongside anomalous videos. Here, anomalies are defined as behaviors that deviate from normal pedestrian activities, such as running, chasing, and excessively fast biking. The task involves detecting “anomalies” without requiring the identification of specific anomalous behavior types. The results of applying the proposed model to this dataset are presented in Table 3, and comparisons with several previous methods demonstrate competitive performance.

For fine-grained anomaly recognition, as illustrated in Table 4 and Table 5, the WSVAD-CLIP model also demonstrates outstanding performance. The approach is compared with previous works such as VadCLIP, AVVD, and Sultani et al. [5,33,40]. The earliest study to propose fine-grained WSVAD, VAAD, was implemented based on CLIP features, while the work by Sultani et al. also involved fine-tuning. As observed, fine-grained WSVAD presents greater challenges compared to coarse-grained WSVAD, as it requires precise multi-class classification and continuous segment-level localization, in contrast to the simpler binary classification setting of coarse-grained detection. In this task, WSVAD-CLIP consistently outperforms prior methods on both the XD-Violence and UCF-Crime datasets. Compared with the work of Sultani et al., substantial gains of 13.66% and 4.46% are achieved on the XD-Violence and UCF-Crime datasets, respectively. On XD-Violence, absolute AVG improvements of 0.61% and 5.1% are obtained over VadCLIP and AVVD, respectively. Similarly, on UCF-Crime, absolute gains of 1.02% over VadCLIP and 1.65% over AVVD are achieved. These results further confirm the effectiveness of the WSVAD-CLIP framework and demonstrate the strong potential of multimodal alignment for improving performance on fine-grained weakly supervised video anomaly detection.

### 4.4. Ablation Studies

In this section, ablation experiments are performed on the UCF-Crime dataset to verify the contribution of each module in the proposed framework.

#### 4.4.1. Effectiveness of AG Module

As shown in Table 6, an ablation study on the AG module is conducted to verify its effectiveness in temporal modeling. Under the baseline setting without any temporal modeling module, the model achieves only 84.57% AUC and 3.82% AVG, indicating that the absence of temporal modeling hinders the ability to capture key abnormal patterns. Next, a Transformer Encoder (TF-encoder) module employing global self-attention [48] is introduced. Although the AUC slightly improves to 85.26%, the AVG remains relatively low at 5.42%, due to the lack of modeling for local temporal dependencies. To enhance local modeling capability, the proposed LiteGAT module is further introduced, which effectively captures local inter-frame structural relations and improves the AUC and AVG to 85.21% and 5.60%, respectively. However, its limited ability to capture global dependencies still leads to some local misjudgments. The Axial Transformer module is then incorporated to strengthen the modeling of long-range dependencies. This results in a significant performance boost, achieving 86.06% AUC and 9.14% AVG, demonstrating strong global contextual modeling capability. Finally, by integrating the strengths of both local and global modeling, LiteGAT is combined with the Axial Transformer to construct the AG Module. This fusion achieves the best performance, with 87.85% AUC and 7.70% AVG, confirming the complementary and synergistic benefits of jointly modeling temporal dependencies at multiple scales.

#### 4.4.2. Effectiveness of Text Prompt

As shown in Table 7, the impact of different text prompt strategies on anomaly detection performance within the WSVAD-CLIP framework is further evaluated. When using static handcrafted prompts as a baseline, the model performance is limited due to the fixed nature of the prompts and lack of semantic adaptability, achieving only 86.32% AUC and 6.30% AVG. To address this, a Learnable Prompt mechanism is introduced, in which a set of trainable context tokens is appended to the original label semantics. This enables the model to optimize the prompt representations in a task-specific manner, thereby guiding the visual encoding process more effectively. This strategy improves performance to 87.75% AUC and 7.34% AVG, demonstrating its advantage in enhancing semantic expressiveness of prompts. Furthermore, a Knowledge-Enhanced Prompt is incorporated by leveraging external knowledge bases such as ConceptNet to construct semantically rich prompt templates. This enhances the model’s understanding of behavioral semantics and results in 86.50% AUC and 7.27% AVG. Although slightly lower than the learnable prompt strategy, this approach shows better generalization in certain categories. Finally, a hybrid prompt strategy is proposed that combines learnable prompt and knowledge-enhanced prompt, integrating the strengths of both task adaptability and semantic prior knowledge. This fusion leads to complementary and reinforced semantic representations, yielding the best performance of 87.85% AUC and 7.70% AVG. These results clearly demonstrate the critical role of prompt design in weakly supervised video anomaly detection.

#### 4.4.3. Effectiveness of Abnormal Visual-Guided Text Prompt

As shown in Table 8, the results of the ablation study on the Abnormal Visual-Guided Text Prompt module are presented. Specifically, the differences in model performance with and without the inclusion of this module are compared. The experimental results show that without the abnormal visual-guided text prompt, the model achieves an AUC of 85.78% and an AVG of 6.12%. After incorporating this module, the performance significantly improves, reaching 87.85% AUC and 7.70% AVG. These results clearly demonstrate that integrating visual context from abnormal segments effectively enhances the quality of category embeddings, improving the model’s ability to perceive and discriminate anomalous events. This, in turn, leads to a notable improvement in overall anomaly detection performance.

### 4.5. Qualitative Results

**Figure 4:**
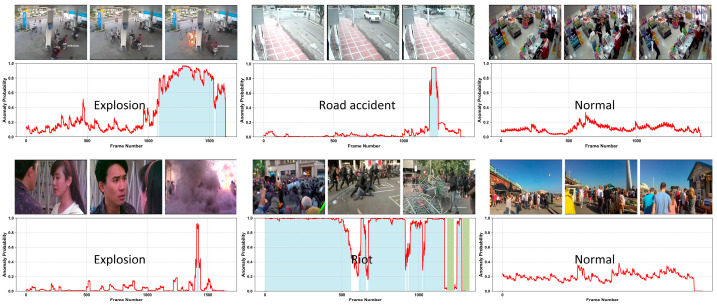
Qualitative results on the UCF-Crime and XD-Violence datasets. The upper row shows visualization diagrams of anomalous and normal frames from UCF-Crime, while the lower row presents the corresponding visualizations for XD-Violence. Blue-shaded regions indicate ground-truth abnormal intervals, and green-shaded regions highlight false positives and false negatives.

Figure 4 illustrates the anomaly detection results on a test video. When an abnormal event occurs, the predicted anomaly scores increase sharply and drop quickly after the event ends, while the scores remain low during normal segments. This behavior demonstrates the model’s strong sensitivity to anomalies and its ability to distinguish normal frames effectively. The figure presents the frame-level anomaly score curve, with selected key frames illustrating the consistency between visual content and the predicted scores. The blue-shaded regions indicate the ground-truth abnormal intervals, while the green-shaded regions highlight false positives (model predicts anomaly but the frame is normal) and false negatives (model fails to detect an actual anomaly). These mispredictions typically occur due to subtle visual changes or ambiguous actions that resemble abnormal events, making it challenging for the model to fully discriminate between normal and anomalous behaviors. Overall, the results demonstrate the effectiveness of the proposed method in accurately and promptly detecting anomalous events.

### 4.6. Discussion

The proposed WSVAD-CLIP framework demonstrates notable performance in weakly supervised video anomaly detection, with 171.59 million parameters, an average inference time of 0.0442 s per frame, and a speed of 22.61 frames per second (FPS).

The AG module, which combines Axial Transformer for global temporal modeling and LiteGAT for local structural awareness, effectively captures abnormal dynamics, with a memory usage of 734.80 Megabytes (MB), a total of 16.55 million parameters, and 4.23 Giga Floating Point Operations (GFLOPs). The average inference time is 0.0137 s per frame, corresponding to 73.21 FPS. Qualitative results show that the anomaly score curves respond sensitively to abnormal events.

The fusion ratio between learnable prompt and knowledge-enhanced prompt has a measurable impact on the results, where an appropriate balance improves the discriminability of class embeddings and visual-semantic alignment; the Text Prompt module, which encodes both types of prompts, requires 766.00 MB of memory, contains 25.20 million parameters, and entails 54.26 GFLOPs per forward pass, highlighting its computational intensity in integrating semantic information.

The AVGTP mechanism further enhances temporal and visual-textual alignment, with a memory footprint of 728.80 MB, 2.10 million parameters, and 0.03 GFLOPs, contributing to improved feature interaction with minimal additional computational cost.

However, some limitations remain. The AVGTP mechanism may overfit because it learns anomaly specific textual prompts, which can memorize training data patterns, especially when datasets are small or imbalanced. The knowledge-enhanced prompt carries a risk of context mismatch, as some concepts retrieved from ConceptNet may not accurately reflect the actual visual content, potentially introducing irrelevant information. Moreover, semantically similar categories (e.g., “Fighting” and “Riot”) and short-duration or highly intertwined abnormal events remain challenging under weak supervision.

Future work may address the overfitting risk of AVGTP, for example, by exploring more robust prompt learning strategies, regularization techniques, or prompt diversification. Efforts may also reduce context mismatch in the knowledge-enhanced prompt, for example, by filtering concepts based on video context, and improve fine-grained anomaly detection, particularly for semantically similar or short/intertwined events. In addition, exploring more efficient temporal modeling and incorporating multimodal information (e.g., audio) could further enhance accuracy and robustness.

## 5. Conclusions

In this study, WSVAD-CLIP is introduced, a novel approach designed for weakly supervised video anomaly detection. To bridge the gap between pre-trained visual features and semantic representations across modalities, an AG Module is designed to enhance the model’s ability to capture long-range temporal dependencies and local motion variations. The module combines an Axial Transformer for global temporal modeling with LiteGAT for localized feature aggregation. This design enhances the model’s sensitivity to abnormal dynamics, although its performance partially depends on the generalization ability of the pre-trained CLIP, which may limit detection in rare or unusual scenarios.

For semantic modeling, a Text Prompt mechanism is integrated that combines a learnable prompt, which introduces trainable context tokens to class labels, with a knowledge-enhanced prompt that leverages external knowledge bases, thereby enriching the semantic representation of class labels and improving visual-semantic alignment. In addition, the proposed AVGPT mechanism further optimizes the textual feature representations by incorporating visual context. Despite these improvements, the model may still have difficulty distinguishing semantically similar anomaly categories, which could slightly affect fine-grained detection. Nonetheless, these innovations collectively improve the model’s perception of dynamic abnormal behaviors and its generalization across diverse scenarios.

Experimental findings confirm that WSVAD-CLIP delivers notable enhancements in performance on both the UCF-Crime and XD-Violence benchmarks. Ablation studies further validate the effectiveness of each module. This framework offers an innovative solution for weakly supervised video anomaly detection. Future work can explore finer-grained modeling of anomaly types, improved cross-modal alignment strategies, and the incorporation of additional modalities such as audio and motion trajectories to enhance the model’s understanding and discrimination of complex anomalous scenarios, thereby further improving detection accuracy and robustness.

## Figures and Tables

**Table 1:** Coarse-grained anomaly detection comparisons on UCF-Crime.

Supervision	Method	Feature	AUC (%)
Semi-supervised	SVM Baseline		50.10
	Hasan et al. [49]		51.20
	BODS [50]	I3D	68.26
	GODS [50]	I3D	70.46
Un-supervised	Zaheer et al. [12]	ResNext	71.04
Fully supervised	Liu&Ma [6]	NLN	82.00
Weakly-supervised	Sultani et al. [5]	C3D	75.41
	GCN [38]	TSN	82.12
	RTFM [16]	I3D	84.30
	MSL [20]	VideoSwin	85.62
	UR-DMU [39]	I3D	86.97
	MGFN [14]	I3D	86.98
	AVVD [40]	CLIP	82.45
	AnomalyCLIP [28]	CLIP	86.36
	OPVAD [47]	CLIP	86.40
	CLIP-TSA [26]	CLIP	87.58
	TPWNG [46]	CLIP	87.79
	WSVAD-CLIP	CLIP	87.85

**Table 2:** Coarse-grained anomaly detection comparisons on XD-Violence.

Supervision	Method	Feature	AP(%)
Semi-supervised	SVM Baseline		50.80
	Hasan et al. [49]		31.25
Weakly-supervised	Sultani et al. [5]	C3D	73.20
	Wu et al. [3]	I3D	73.20
	RTFM [16]	I3D	77.81
	MSL [20]	VideoSwin	78.58
	UR-DMU [39]	I3D	81.66
	MGFN [14]	VideoSwin	80.11
	AVVD [40]	CLIP	78.10
	AnomalyCLIP [28]	CLIP	78.51
	OPVAD [47]	CLIP	66.53
	CLIP-TSA [26]	CLIP	82.19
	TPWNG [46]	CLIP	83.68
	WSVAD-CLIP	CLIP	83.95

**Table 3:** Coarse-grained anomaly detection comparisons on ShanghaiTech.

Supervision	Method	Feature	AUC(%)
Semi-supervised	Zaheer et al. [12]	ResNext	79.62
Un-supervised	Zaheer et al. [12]	ResNext	78.93
Weakly-supervised	GCN [38]	TSN	84.44
	Sultani et al. [5]	CLIP	91.72
	Wu et al. [3]	CLIP	95.24
	SSRL [13]	CLIP	96.22
	RTFM [16]	CLIP	96.76
	OPVAD [47]	CLIP	96.98
	MSL [20]	VideoSwin	97.20
	WSVAD-CLIP	CLIP	97.31

**Table 4:** Fine-grained anomaly recognition comparisons on XD-Violence.

Method	mAP@IOU(%) AVG
Random Baseline	0.71
Sultani et al. [5]	11.65
AVVD [40]	20.21
VadCLIP [33]	24.70
WSVAD-CLIP	25.31

**Table 5:** Fine-grained anomaly recognition comparisons on UCF-Crime.

Method	mAP@IOU(%) AVG
Random Baseline	0.08
Sultani et al. [5]	3.24
AVVD [40]	6.05
VadCLIP [33]	6.68
WSVAD-CLIP	7.70

**Table 6:** Effectiveness of AG Module.

Method	AUC (%)	AVG (%)
Baseline (without temporal modeling)	84.57	3.82
TF-encoder	85.26	5.42
Only GAT	85.21	5.60
Axial Transformer	86.06	9.14
AG Module	87.85	7.70

**Table 7:** Effectiveness of Text Prompt.

Method	AUC (%)	AVG (%)
Hand-crafted Prompt	86.32	6.30
Learnable Prompt	87.75	7.34
Knowledge-Enhanced Prompt	86.50	7.27
Learnable+ Knowledge Enhanced (Text Prompt)	87.85	7.70

**Table 8:** Effectiveness of AVGTP.

Method	AUC (%)	AVG (%)
w/o AVGTP	85.78	6.12
w AVGTP	87.85	7.70

## Data Availability

The original contributions presented in this study are included in the article. Further inquiries can be directed to the corresponding author.
